# Resting motor threshold and magnetic field output of the figure-of-8 and the double-cone coil

**DOI:** 10.1038/s41598-020-58034-2

**Published:** 2020-02-03

**Authors:** Martin Schecklmann, Maximilian Schmaußer, Felix Klinger, Peter M. Kreuzer, Lars Krenkel, Berthold Langguth

**Affiliations:** 1 Department of Psychiatry and Psychotherapy, University of Regensburg, Regensburg, Germany; 2 Fakultät Maschinenbau, Ostbayerische Technische Hochschule, Regensburg, Germany

**Keywords:** Motor cortex, Human behaviour

## Abstract

The use of the double-cone (DC) coil in transcranial magnetic stimulation (TMS) is promoted with the notion that the DC coil enables stimulation of deeper brain areas in contrast to conventional figure-of-8 (Fo8) coils. However, systematic comparisons of these two coil types with respect to the spatial distribution of the magnetic field output and also to the induced activity in superficial and deeper brain areas are limited. Resting motor thresholds of the left and right first dorsal interosseous (FDI) and tibialis anterior (TA) were determined with the DC and the Fo8 coil in 17 healthy subjects. Coils were orientated over the corresponding motor area in an angle of 45 degrees for the hand area with the handle pointing in posterior direction and in medio-lateral direction for the leg area. Physical measurements were done with an automatic gantry table using a Gaussmeter. Resting motor threshold was higher for the leg area in contrast to the hand area and for the Fo8 in contrast to the DC coil. Muscle by coil interaction was also significant providing higher differences between leg and hand area for the Fo8 (about 27%) in contrast to the DC coil (about 15%). Magnetic field strength was higher for the DC coil in contrast to the Fo8 coil. The DC coil produces a higher magnetic field with higher depth of penetration than the figure of eight coil.

## Introduction

Transcranial magnetic stimulation (TMS) has traditionally been considered to be a method that enables the direct modulation of neuronal activity in superficial cortical areas, but not in deeper brain areas, as the strength of the produced magnetic field strongly declines with increasing distance from the coil. Activity changes in more distant areas were explained as propagation of stimulation effects from the directly stimulated area to functionally connected remote brain areas^1–4^. More recently new coil geometries (double-cone coil, batwing coil, H-coil) have been developed, which differ in the spatial configuration of the produced magnetic field and enable direct stimulation of skull-distant areas^5,6^. This is for example of high relevance in treatment of depression with repetitive TMS (rTMS). One core region in the aetiology of affective and other neuropsychiatric disorders is the anterior cingulate cortex^7,8^. Moreover, after rTMS treatment in depressive patients structural and functional changes were observed in prefrontal and cingulate cortex^9^. In line with this, the pre-treatment activity of the anterior cingulate turned out to be a positive predictor for treatment response in pharmacological^10^ and rTMS^11^ treatment of depression.

Thus, direct stimulation of the cingulate cortex may be a good alternative for rTMS treatment in affective disorders or other neuropsychiatric conditions. The double-cone (DC) coil with its angled shape is predicted to reach deep brain areas as claimed by information from TMS manufactures (www.magstim.com; www.magventure.com) and simulation studies^6,12^. Behavioural^13^, neuroimaging^14,15^ and clinical studies^16–19^ suggest that the DC coil indeed modulates cingulate or medial prefrontal cortex function even if it is still under debate whether these regions are reached directly or transsynaptically via stimulation of superficial brain areas.

Most of the mentioned studies did not directly compare rTMS with a conventional Fo8 coil and rTMS with the DC coil except in two clinical studies of depression and tinnitus^18,19^ and one investigating the hand motor threshold in healthy subjects^20^. The available clinical pilot studies from our working group suggest faster effects^18,19^ of rTMS using the DC coil, but the data are still very preliminary. Hand motor threshold was lower for the DC coil in contrast to the Fo8 coil^20^. Furthermore, we are not aware of one single study measuring the induced magnetic field of both coils in a technical examination.

Reviewing the methods for determining stimulation intensity of studies using the DC coil it turned out that there is high heterogeneity^21^. In some studies the intensity of DC coil stimulation was chosen according to motor thresholds determined with the figure of eight coil, others determined the motor threshold with the DC coil, but sometimes by stimulating the hand area and sometimes by stimulation of the leg area^21^. Several studies also used fixed stimulator intensities independent of the individual motor threshold^21^. Additionally there were also differences in coil positioning and orientation^21^. Some parameters were not reported at all^21^.

In sum, there is limited information if TMS-induced activity in the hand area can be transferred or can be equated to physiological actions in the leg area. There is also a lack of evidence of whether the Fo8 and DC coils are acting in the same physiological way. There is even no evidence from physical measurements if the output of the coils is comparable. Thus, the aim of the present work was to answer two basic questions about the magnetic field produced by the DC coil in comparison to the Fo8 coil. How do the two coils differ in their ability to stimulate superficial and deeper brain areas? What is the difference in the spatial distribution of the magnetic field strength between the two coils equated in machine output? For this purpose we determined the resting motor thresholds of the left and right first dorsal interosseous (FDI) and tibialis anterior (TA) “*in vivo*” with the two coils. In a separate “*in vitro*” experiment we measured the induced magnetic field of both coils with an automatic gantry table using a Gaussmeter.

## Results

### Resting motor threshold

**Figure 1:**
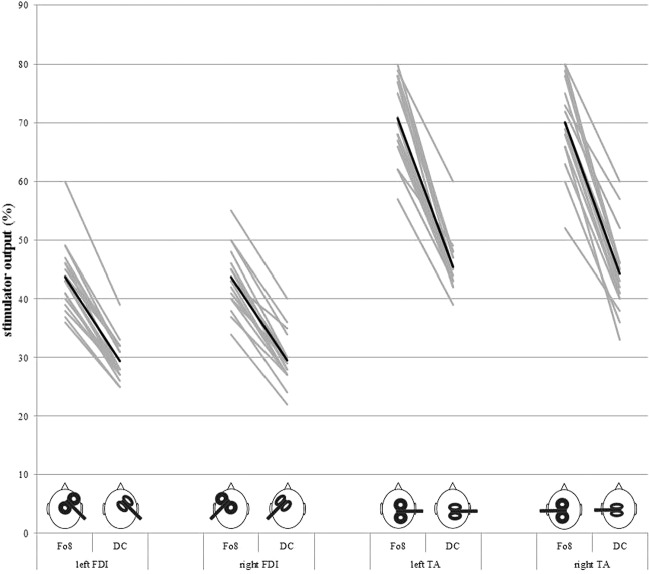
Resting motor threshold as indicated by stimulator output necessary to reach RMT in dependence from coil type (figure of eight (Fo8), double-cone (DC)), target muscle (first dorsal interosseous (FDI), tibialis anterior (TA)) and stimulated side of the brain (left vs. right). Single values in grey, average value in black. Coil positioning and orientation is shown on the bottom.

The three-way ANOVA revealed two significant main effects for the factor muscle (F = 357.3, df = 1,16; p < 0.001; η_p_² = 0.957) and coil (F = 688.17; df = 1,16; p < 0.001; η_p_² = 0.977) which indicated higher RMTs for the TA in contrast to the FDI muscle and for the Fo8 in contrast to the DC coil (Fig. 1). There was also a significant interaction between muscle and coil (F = 62.86; df = 1,16; p < 0.001, η_p_² = 0.797). RMT differences between the Fo8 and DC coil were higher for the TA (about 26%) in contrast to the FDI (about 14%). RMT difference between the TA and the FDI muscle was higher for the Fo8 (about 27%) in contrast to the DC coil (about 15%). Main or interaction effects of side were not significant (all F-values < 0.542; df = 1,16; all p-values > 0.472). Mean correlation coefficient for the association between left and right cortex was 0.703 (all p-values < 0.004), for the association of hand and leg area 0.454 (all p-values < 0.256) and for the association of the Fo8 and DC coil 0.730 (all p-values < 0.035).

### Induced magnetic field

**Figure 2:**
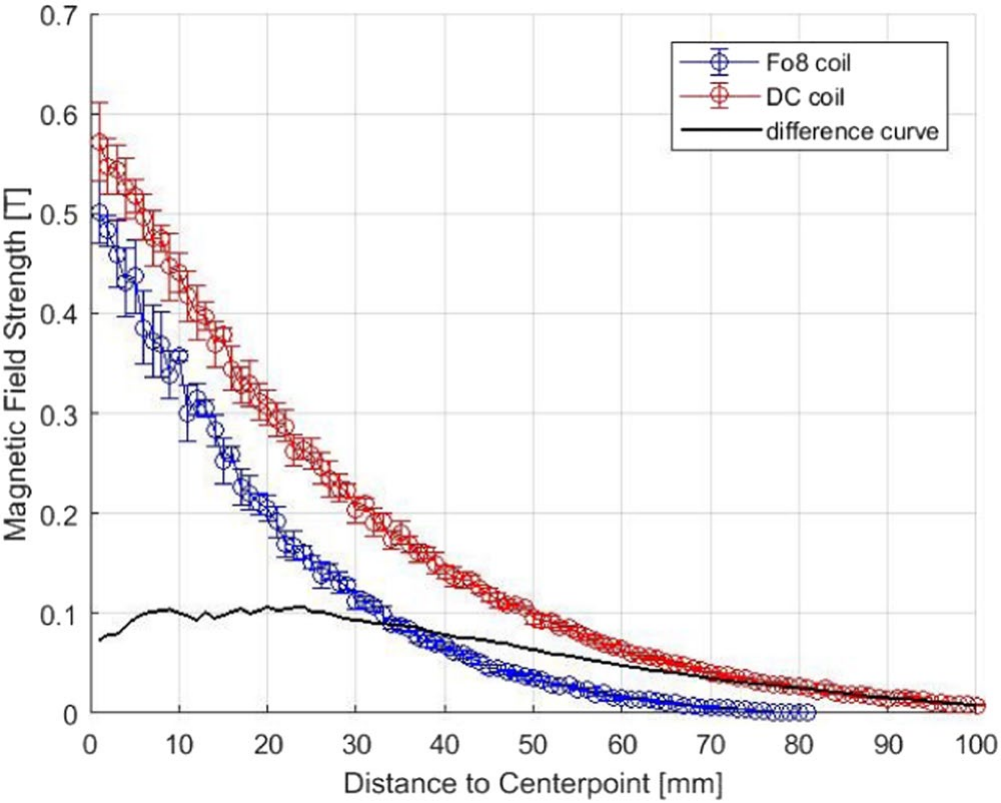
Induced magnetic field strength of monophasic pulses of 100% stimulator output in dependence from distance to the center of the coil measured along an orthogonal line for a figure of eight (C-B60) and a double-cone coil (D-B80). The difference curve of both coils was shown with a moving average of 10 mm.

Figure 2 indicates the dependence of the magnetic field strength from distance to the center of the coil in orthogonal direction. Magnetic field decreased in an exponential decay for both coils with higher output for the DC coil in contrast to the Fo8 coil with approximating magnetic fields with increases of the measurement position to the coil.

## Discussion

In summary, the DC coil induces higher magnetic fields in contrast to the Fo8 coil. In accordance, RMTs were higher for the Fo8 coil in contrast to the DC coil. Furthermore, RMTs were higher for the leg (i.e. deeper) area in contrast to the hand (i.e. coil-near) area.

Systematic comparisons of resting motor threshold (RMT) of side of stimulation (left vs. right), target muscle area (hand vs. leg) and coil type (Fo8 vs. DC) revealed higher RMT for the leg area as measured at the TA in contrast to the hand area as measured at the FDI. This was expected as the leg area is deeper lying (in the depth of the superior sagittal sinus) in contrast to the hand area (superficial skull-near precentral gyrus) which means that the distance from the center of the coil is larger for the leg area. Considering that the cingulate cortex as potential target area for therapeutic rTMS is lying even deeper in the brain than the leg area, even higher stimulation intensities might be required to reach the cingulate or mediofrontal cortex directly. Considering in this context that higher stimulation intensities come along with higher probability for side effects and reduced tolerability it is necessary to find the adequate balance between treatment efficacy on one side and subject comfort on the other side.

Another finding is that the RMT is higher for the Fo8 coil in contrast to the DC coil for the hand and the leg area. This was already shown for the hand area^20^ and fits with our physical measurements which showed that the magnetic field is stronger for the DC coil both directly below the coil and also with increasing distance to the coils. Also simulation studies pointed out the higher intensity of the DC coil type^12^. One aspect which might influence the physical properties of the induced magnetic field beside shape and should be addressed in future studies is the difference in coil diameter between the DC and the Fo8 coil.

A further main finding of our study was a muscle by coil interaction effect. The increased RMT for the Fo8 coil in contrast to the DC coil was higher for the leg in contrast to the hand area. This means that the advantage of the DC coil is more pronounced for the deeper leg area as compared to the superficial hand areas. Two different explanations may account for the finding. First, this finding could reflect the slightly slower decline of the magnetic field of the DC coil with increasing distance from the coil. An alternative explanation might be the lower focality of the DC coil. The stronger magnetic field of the DC coil is associated with reduced focality^22^ which makes it easier to hit deeper lying brain areas more exactly. For stimulation of superficial brain areas the focality of the magnetic field plays a minor role as compared to the stimulation of deeper targets as the target immediately below the coil is still reached even if the coil orientation or rotation is changed. This is in line with the findings of the correlation analyses which showed higher associations for left vs. right and for Fo8 and DC coil in contrast to the association for hand vs. leg area. The correlations were high for associations that did not contrast coil-near and deep areas (hand vs. leg area) and highlights that variability is increased for comparison of hand and leg area in contrast to left and right side or coil type.

We want to stress that we had to stop measurements in three participants because of vasovagal syncopes (stimulation of the leg area with the DC coil) or dizziness (stimulation of the leg area with the Fo8 coil). So far it was reported that stimulation with the DC coil is well-tolerated. This high drop-out rate highlights the need for careful handling of and systematic recording of side effects. Side effects might emerge especially for high stimulation intensities as it is necessary for the leg area and as it is the case for stimulation with the DC coil which might be perceived as more aversive^19^. The stimulated peripheral skin and muscle area on the head is higher for the DC coil used in this study due to the larger diameter and the angulation of the two wings. The strength of the produced magnetic field (see physical measurements) is also higher for the DC coil and finally the stimulation of deeper areas is associated with higher intensities and lower focality of the magnetic field which is known as depth-focality trade-off^22^ probably inducing more side effects.

## Conclusions

For the first time, we contrasted the Fo8 and DC coil with respect to magnetic field output and RMTs in the hand and leg area for different stimulation sides and could demonstrate that the DC coil induces higher magnetic fields in deeper brain areas which went along with reduced RMTs in coil-near and more distant areas. The advantage of the DC coil was more pronounced in the stimulation of the deeper leg area as compared with the more superficial hand area. Stimulation of left or right motor cortex showed no differences. Not investigated parameters but variables of interest for future studies are even deeper lying brain areas which can be measured with stimulation of foot muscles, coil orientation for the TA (handle pointing towards the ears vs. towards the nose), the electric output instead of the magnetic field and also of the induced field in brain tissue of cadavers.

## Methods and Materials

### Participants

We recruited 22 healthy individuals without any records of psychiatric or neurological disorders, who fulfilled the safety criteria for TMS. Each participant was tested in an individual session and was compensated with 20€. Five persons were excluded from the final analysis. Two subjects had vasovagal syncopes during stimulation of the leg area with the DC coil and one dizziness during stimulation of the leg area with the Fo8 coil. Two other participants were excluded because of incomplete measurements due to high motor thresholds exceeding safety limits. For safety reasons we allowed maximal stimulation intensities of 80% stimulator output for the Fo8 coil and of 60% for the DC coil. The final sample comprised 17 individuals, aged 20 to 25 (22.59 ± 1.32, 41% female). All the included participants were right-handed as assessed with the Edinburgh Handedness Inventory^23^. Written informed consent was obtained from each subject. The study protocol was approved by the local ethics committee (University of Regensburg; number 16-101-0038) and the used methods were carried out in accordance to the latest version (5^th^ revision) of the Declaration of Helsinki.

### Procedures of physiological measurements

The study was performed at the Department of Psychiatry and Psychotherapy of the University of Regensburg in November and December 2016. Participants underwent a screening interview for personal data, medical history and consumption habits to screen for contraindications. After that, they filled in various questionnaires to screen for depression (Major Depression Inventory), intelligence (multiple-choice vocabulary intelligence test) and handedness (Edinburgh Handedness Inventory)^23–25^. Participants were not depressed and showed normal to high intelligence. Then, participants were seated comfortably in a chair and a tight fitting cap was placed on each subject’s head. To identify the areas of stimulation, the vertex was marked on this cap by measuring the mid-point intersection between the nasion-inion and inter-aural lines. Recording surface electromyography (EMG) electrodes were placed on left and right first dorsal interosseous (FDI) and tibialis anterior (TA). Next, resting motor thresholds (RMT) for all four areas were determined with both a C-B60 Butterfly Coil and a D-B80 Butterfly Double-Cone-Coil (MagVenture A/Sm, Denmark). For the FDI, the respective coil was positioned on the left or right hand motor cortex with the coil handle positioned at an angle of 45° to the midline pointing backwards. For the TA, the respective coil was placed 1–2 cm posterior to the vertex and 1–2 cm laterally to the left or to the right, with the current flow directed towards the hemisphere to be stimulated. The coil position was adjusted for each individual to the site that elicited motor evoked potentials (MEPs) with maximal amplitude in the resting target muscle. RMT was defined as the lowest stimulation intensity which produced in 4 out of 8 trials a motor evoked potential of at least 50 µV. The sequence of the stimulated areas and of the coil types was randomized.

A three-way repeated measures ANOVA with the factors muscle (TA vs. FDI), coil (Fo8 vs. DC) and side (right vs. left) and the dependent variable RMT was conducted. The ANOVA was done to analyse differences in the strength of the induced physiologic activity. To investigate the similarity of physiologic activity of left and right hemisphere, of both coil types and both muscles we did also correlation analyses (left vs. right hand for the Fo8 and DC coil, left vs. right leg for the Fo8 and DC coil; hand vs. leg area for the left and right cortex for the stimulation with the Fo8 and the DC coil; Fo8 vs. DC coil for the left and right hand and for the left and right leg area). All four correlations were averaged.

### Physical measurements: induced magnetic field

**Figure 3:**
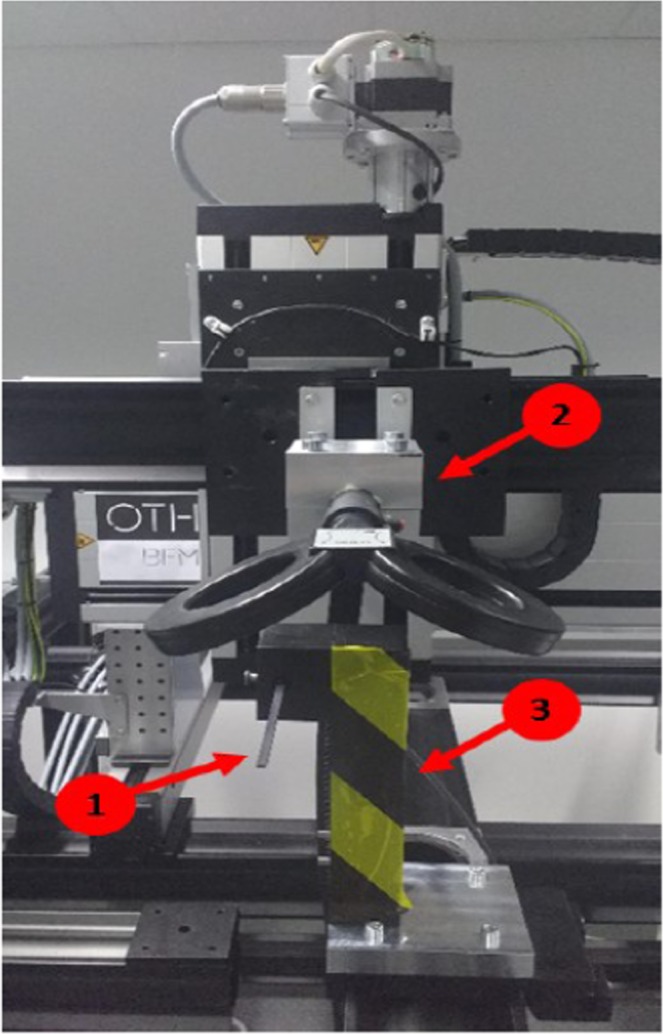
Setup for the physical measurements - exemplarily for the DC coil. The induced field was measured for both coils using a transverse Hall sensor (1). Sensor holding (3) and coil holding (2) were mounted on a positioning unit of an automatic gantry table.

The induced field was measured for both coils using a transverse Hall sensor (HMMT-6J04-VF magnetic field probe, Lakeshore Cytrotronics, USA) (Fig. 3). The magnetic field probe consists of a 103 mm long aluminum shaft that rises from a plastic stem. Magnetic field measurements take place in the sensing area of the probe, which is located 4 mm from the end of the shaft and has a width of 5 mm. The holding for the magnetic field probe was made of Polyoxymethylene (POM) in order to prevent a distortion of the field. Sensor holding and coil holding were mounted on a positioning unit of an automatic gantry table (iMC-S8-controller; isel Germany AG, Germany) with a step motor with an accuracy of 0.006 mm. Magnetic field values were obtained using a DSP 455 Gaussmeter (Lakeshore Cytrotronics, USA). Measurement mode of the Gaussmeter was set to peak mode, which allows for the detection of pulsed magnetic fields with a pulse width of 50 µs or greater. The synchronization of coil movement, stimulus delivery and data acquisition was done with LabVIEW (National Instruments, USA).

The probe was positioned directly below the center of the coils and the surface of the coil was parallel to the sensing area of the probe. Planar coil-probe orientation is essential because probe recordings are dependent upon the angle of the Hall sensor relative to the magnetic field. Maximum output occurs when the flux vector is perpendicular to the plane of the sensor. Measurements were done in 1 mm steps in orthogonal angle to the plane of the coil starting with a start distance of 1 mm to the coil. Please note that we did not obtain the value of the three-dimensional field for which it would have been necessary to conduct one separate measurement for each of the principal planes. For physical measurements we used a monophasic pulse form with a stimulation intensity of 100% of stimulator output. For each position three pulses were measured and averaged.

## Data Availability

The datasets generated during and/or analysed during the current study are not publicly available due to restrictions by our ethics committee but are available from the corresponding author on reasonable request.
